# Cis-4-[18F]fluoro-L-proline PET/CT molecular imaging quantifying liver collagenogenesis: No existing fibrotic deposition in experimental advanced-stage alcoholic liver fibrosis

**DOI:** 10.3389/fnume.2022.952943

**Published:** 2022-08-23

**Authors:** Na Duan, Hongxia Chen, Liya Pi, Youssef Ali, Qi Cao

**Affiliations:** ^1^Affiliated Hospital of Nanjing University of Chinese Medicine, Nanjing, China; ^2^School of Medicine, University of Maryland, Baltimore, MD, United States; ^3^University of Maryland Medical Center, Tulane University, New Orleans, LA, United States

**Keywords:** alcoholic, PET/CT, cis-4-[18F]fluoro-L-proline, hepatic stellate cells, liver fibrosis

## Abstract

**Background and purpose:**

Heavy alcohol drinking-induced alcoholic fatty liver, steatohepatitis, and early-stage alcoholic liver fibrosis may progress to advanced-stage alcoholic liver fibrosis (AALF)/cirrhosis. The lack of non-invasive imaging techniques for the diagnosising collagenogenesis in activated hepatic stellate cells (HSCs) can lead to incurable liver fibrosis at the early reversible stage. Proline has been known as the most abundant amino acid of collagen type 1 synthesized by activated HSC with the transportation of proline transporter. *cis*-4-[^18^F]fluoro-L-proline ([^18^F]proline) was reported as a useful tool to quantify collagenogenesis in experimental alcoholic steatohepatitis. This study aims to use [^18^F]proline micro PET as non-invasive imaging to quantify liver collagenogenesis in HSC of experimental AALF.

**Methods:**

AALF model was set up by a modified Lieber-DeCarli liquid ethanol diet for 12 weeks along with intraperitoneal injection (IP) of CCl_**4**_ (0.5 ml/kg) between the 5th and 12th weeks. Controls were fed an isocaloric liquid diet and IP. PBS. *In vitro* [^3^H]proline uptake by HSCs isolated from livers was quantified using a liquid scintillation counter. Collagen type 1 production in HSCs culture medium was assayed by ELISA. *Ex vivo* liver collagen type 1 and proline transporter protein were compared between AALF rats (*n* = 8) and mice (*n* = 8). [^3^H]Proline uptake specificity in *ex vivo* liver tissues was tested using unlabeled proline and transporter inhibitor benztropine at different doses. Liver H&E, trichrome stain, and blood biochemistry were tested in rats and mice. *In vivo*, at varying times after instillation, dynamic and static [^18^F]proline micro PET/CT were done to quantify tracer uptake in AALF mice (*n* = 3). Correlation among liver collagen, liver SUVmax, normalized liver-to-brain ratio, normalized liver-to-thigh ratio, and fluoro-proline-induced collagen levels in *ex vivo* liver tissues were analyzed.

**Results:**

*In vitro* HSCs study showed significant higher [^3^H]proline uptake (23007.9 ± 5089.2 vs. 1075.4 ± 119.3 CPM/mg, *p* < 0.001) in HSCs isolated from AALF rats than controls and so was collagen type 1 production (24.3 ± 5.8 vs. 3.0 ± 0.62 mg/ml, *p* < 0.001) in HSCs culture medium. Highly positive correlation between [^3^H]proline uptake and collagen type 1 by HSCs of AALF rats was found (*r* value = 0.92, *p* < 0.01). *Ex vivo* liver tissue study showed no significant difference in collagen type 1 levels between AALF rats (14.83 ± 5.35 mg/g) and AALF mice (12.91 ± 3.62 mg/g, *p* > 0.05), so was proline transporter expression between AALF rats (7.76 ± 1.92-fold) and AALF mice (6.80 ± 0.97-fold). Unlabeled fluoro-proline induced generation of liver tissue collagen type 1 and [^3^H]proline uptake were specifically blocked by transporter inhibitor. *In vivo* [^18^F]proline micro PET/CT imaging showed higher SUVmax in liver (4.90 ± 0.91 *vs*. 1.63 ± 0.38, *p* < 0.01), higher normalized liver/brain ratio (12.54 ± 0.72 vs. 2.33 ± 0.41, *p* < 0.01), and higher normalized liver/thigh ratio (6.03 ± 0.78 vs. 1.09 ± 0.09, *p* < 0.01) in AALF mice than controls, which are all positively correlated with fluoro-proline-induced levels of collagen in liver tissue (*r* value ≥ 0.93, *p* < 0.01) in AALF mice, but not correlated with existing liver collagen. Liver histology showed increased collagen in the liver of AALF mice. Blood serum ALT and AST levels were remarkably higher in AALF mice than in controls, but there is no significant difference in blood fibrotic parameters HA, A2M, TGFβ1, and MMP1.

**Conclusions:**

[^18^F]proline micro PET/CT might be useful to visualize collagenogenesis in activated HSC of experimental AALF but fails to quantify existing liver collagen in AALF mice. [^18^F]proline has the potential sensitivity to assess the activity and severity of liver fibrosis.

## Introduction

Excessive alcohol consumption is one of the leading causes of advanced-stage liver disease, ranging from simple steatosis, liver inflammation, and liver fibrosis to severe forms of liver damage such as liver cirrhosis and hepatocellular carcinoma (1). Assessing the degree of liver fibrosis in alcoholic liver disease has diagnostic and prognostic implications. Liver fibrosis can potentially regress from an advanced stage to an early stage or even reverse to a normal architecture (2). The gold standard in assessing liver fibrosis is a biopsy. However, biopsy does not show sample differences in disease across the entire liver and suffers from inter-observer variability and patient compliance (3). Repeated biopsies to assess disease progression or therapy response are unattractive due to the increased risk of complications (4). Therefore, there exists an unmet medical need for a non-invasive technique that can be used to identify liver fibrosis progression as well as to monitor response to treatment in patients.

Non-invasive laboratory and imaging techniques have been developed as potential alternative methods for diagnosing liver fibrosis. Panels of blood-based biomarkers, such as FibroTest and the enhanced liver fibrosis (ELF) panel of extracellular matrix (ECM) markers, have been developed as predictors of liver fibrosis but cannot reliably differentiate stages of fibrosis (1, 4). Transient ultrasound elastography (FibroScan) is an imaging-based diagnostic test of the liver. It has been reported with excellent diagnostic accuracy for diagnosing liver fibrosis (stiffness and scarring). However, for the diagnosis of earlier fibrosis stages, high variability was found that is dependent on the underlying liver disease and inter-observer variation (5, 6). MR elastography and collagen-enhanced MRI method could be performed alongside these other tests to diagnose liver fibrosis more accurately (7, 8). Unfortunately, conventional imaging was not powered to stage liver fibrosis at a mild to the moderate stage and required further evaluation and optimization.

Liver fibrosis is characterized by excess deposition of extracellular matrix proteins (mainly type I collagen) (9, 10). In an animal model, carbon tetrachloride (CCl4) is classic toxicity that causes liver fibrosis (11). As fibrosis progresses, collagen deposition and its extracellular location make it readily accessible to the biomarker. Recent studies evaluated the clinical value of PET/CT in the diagnosis of liver fibrosis in patients and animal models (12, 13). In another study, [^18^F]MAGL-4-11 demonstrated reduced PET signals in the early stages of fibrosis and further significantly decrease with disease progression compared with control mice (14). Previously, we identified a [18F]proline that could serve as a PET imaging biomarker for detecting early-stage liver fibrosis in an LPS-induced mice model (15). That study was not powered to stage liver fibrosis as the animals were only imaged at an early-stage time point. Here, we performed [^18^F]proline PET/CT imaging to quantify liver collagenogenesis in experimental mice of advanced stage. We hypothesized that a [^18^F]proline PET/CT molecular imaging measuring collagen levels of advanced liver fibrosis might have broad utility in staging liver fibrosis.

## Materials and methods

### Animals

All animals were cared for following the approved Institutional Animal Care and Use Committee. Experimental imaging procedures and radiotracer operation protocols were approved by the Radiation Safety Operation Committee of the University of Maryland School of Medicine. AALF rats and their controls were used for *in vitro* and *ex vivo* experiments. AALF mice and their controls were used for *ex vivo* and *in vivo* experiments.

### *In vitro* experiments

Sprague-Dawley (SD) rats (20–27 weeks old, 353 ± 58 g body weight, female) were purchased from Charles River Laboratories (Wilmington, MA, United States). They were housed in the University of Maryland School of Medicine. AALF model was induced in eight rats by feeding liquid Lieber-De Carli ethanol diet (# D710260 and D710027, Dyets, USA) at a dose of 2.5 g/kg body weight for 12 weeks along with intraperitoneal injection (IP) of carbon tetrachloride (CCl4) (Sigma, St Louis, MO, United States) at a dose of 0.5 mg/kg body weight two times every week during the 5–12th weeks. Eight normal female Sprague-Dawley (SD) rats were fed an isocaloric liquid diet. They were intraperitoneally injected with PBS (#70011069, Thermo Fisher, USA) at a dose of 0.5 mg/kg body on the same schedule as the AALF rats. Rat livers yield significantly more HSCs than mouse livers. The tritiated position of [^3^H]proline (PerkinElmer, Melville, NY) is L-[2,3-3H]-Proline, and the molar activity at the point of use is 55 to 85 Ci/mmol. [^3^H]proline at the dose of 37 MBq/L was added to cultures under an atmosphere of 95% O_2_ and 5% CO_2_ at 37°C for up to 90 min. Hepatocyte, Kupffer cells, and HSC were isolated from AALF and control rats were cultured in a medium for 12 h and then along with 37 MB g /L of [^3^H]proline cultured for up to 120 min. The uptake of [^3^H]proline was measured by a liquid scintillation counter (PerkinElmer, Shelton, CT). After [^3^H]proline treatment, the culture medium was removed, and cells were washed with 5 ml cold HBSS. The cells were lysed with 0.5 mL 0.5% Triton X-100. Cell lysates were scraped from the plate wells and collected in 1 ml tubes. After the samples were centrifuged at 12,000 rpm at four degrees for 10 min, 125 ml of suspended lysate was transferred into a 5-mL scintillation cocktail in a plastic scintillation tube and measured on a scintillation counter. Data were expressed as counts per minute (CPM)/mg protein. Collagen type 1 production in HSC culture medium from AALF rats and controls was assayed by enzyme-linked immunosorbent assay (ELISA) (16). We evaluated [^3^H]proline uptake, collagen type 1 levels, and mRNA expression of α1(1) procollagen type 1 measured in HSCs *in vitro*. Then, we measured [^3^H]proline uptake by HSC, hepatocytes, and Kupffer cells derived from AALF and control SD rats.

### *Ex vivo* experiments

This AALF mouse model was used to test the specificity of [^3^H]proline uptake by HSC within the liver. Eight C57BL/6 mice (aged 6 to 8 weeks, 15-20 g body weight) were fed Lieber-De Carli ethanol liquid diets at a dose of 2.5 g/kg for 12 weeks. During the 5–12th weeks before the *ex vivo* experiments, the mice were administered CCl4 injections (0.5 mg/kg body weight intravenously). Controls were fed an isocaloric liquid diet on the same schedule. Slices of 8 mm diameter and 300 μm thickness were prepared and cultivated from 100 mg wet weight liver slices of AALF mice and corresponding controls in six-well culture plates. Liver slices were preincubated for 1 h at 37°C in Williams' Medium E with 2 mM Glutamax-I, 25 mM D-glucose, and 50 mg/ml gentamycin under an atmosphere of 95% O_2_ and 5% CO_2_, while gently shaken (70 times/min). After that, slices were transferred to 25 ml tissue culture flasks, then added unlabeled fluoro-proline/proline transporter inhibitor in different doses, and incubated for 90 min, at 37°C for 16 h. [^3^H]Proline in the liver slices of CCl4-treated rats was measured following our previous methods (17). RT-PCR experiments were performed to demonstrate the expression of the proline transporter in rats and mice. Proline transporter protein expression was assessed using a polyclonal antibody against the proline transporter at a dilution of 1:200 (Fisher, Waltham, MA, United States).

Body weight and liver weight in AALF mice and controls were measured. Hematoxylin and eosin staining based on standard Elastica-van Gieson protocol was performed to evaluate overall liver architecture in AALF SD rats, mice, and controls. Liver sections were stained with Masson's trichrome stain to evaluate fibrosis using a Nikon microscope (YTHS, Japan) at 60x magnification. Two pathologists reviewed all images independently through blinded assessment according to the Kleiner scoring system as described in previous studies [steatosis (0–3), lobular inflammation (0–3), fibrosis scores (0–3), and ballooning (0–2)] (18). Blood serum levels of alanine aminotransferase (ALT), aspartate aminotransferase (AST), hyaluronic acid (HA), alpha-2 macroglobulin (A2M), and matrix metalloproteinase 1 (MMP1) concentrations in the serum of AALF mice and controls were measured by commercial kits. Total transforming growth factor-beta 1 (TGF-β1) protein was detected on tissue sections of AALF mice and controls using immunohistochemistry (S-P).

### *In vivo* experiments

The mouse model was used for *in vivo* experiments because of the strikingly similar experimental results compared with the rat model, such as representative liver morphology, collagen type 1, and proline transporter protein levels in livers. However, it reduces the amount of imaging radiotracer doses and feeding costs. To study the correlation between liver collagenogenesis under different conditions and the progression of liver fibrosis, [^18^F]proline PET/CT imaging (Siemens Inveon Small Animal PET/CT Imaging System, Siemens) was performed in anesthetized mice (*n* = 6, three mice in the AALF group and three in the control group). Afterward, PET/CT imaging was handled at the Core for Translational Research in Imaging at the University of Maryland School of Medicine. Six female C57BL-6 mice were injected with 8 ± 1 MBq of [^18^F] Proline *via* a tail vein catheter. Images were performed 60 min after the solution was administrated, and total imaging length was acquired for 30 min. Static [^18^F]proline PET imaging was obtained during the 90–120 min period, respectively, with no other tracer injection. In the next place, to obtain liver HSC activation by static [^18^F]proline imaging, the body weight of the AALF and control mice were measured and up to 11 MBq in 200 ml of [^18^F]proline at the same proline concentration of 19 mmol/L was injected via a tail vein catheter. In the end, static PET imaging achievement for liver HSC activation was performed over 30 min and 60 min after administration of [^18^F]proline.

Subsequently, imaging acquisition with a CT scan was used for attenuation correction and localization of the lesion site. Multiplanar reformatted views of PET/CT images in the sagittal, coronal, and trans axial planes were performed to help with anatomical co-localization and segmentation. The radiotracer biodistribution in 12 critical organs, including the liver, lung, pancreas, brain, heart, spleen, stomach, colon, thigh, spine cord, kidney, and bladder, were quantified by regions of interest (ROI) segmentation integrations. The liver is centered in the field of view. The images were evaluated by ROI placed on the margin of the liver and a corresponding region set on the area of brain and thigh tissue. PET data of tracer uptake were calculated in megabecquerel per liter (MBq/L) and injected dose (%ID/g) of targeted organs. Data analyses of imaging were performed using the commercially available InveonTM Research Workplace PET image analysis software (Inveon PET, Siemens Medical Solutions, PA). Mean radioactivity uptake within each ROI was measured to quantify the uptake of [^18^F]proline. Standardized uptake value (SUV) is used as a parameter of the semi-quantitative analysis.

### Statistics

Statistical analyses were carried out using the Graphpad Prism 9.0 software (Graphpad Software, LaJolla, California, USA). Each experiment was carried out with cells from at least three donors (*n* = 3), if not stated differently. One author entered the original data, and the other author checked data consistency and integrity. The continuous variables were used, Student's *t*-test and the Manne–Whitney U-test, as appropriate. The Analysis of Variance (ANOVA) or the Kruskal–Wallis test was used to compare a variable among three or more groups. The correlation between two variables was quantified by Pearson correlation coefficients (r). Variables with a *p*-value < 0.05 were considered statistically significant.

## Results

### *In vitro* [^3^H]proline uptake and collagen type 1 levels in AALF rats and controls

**Figure 1:**
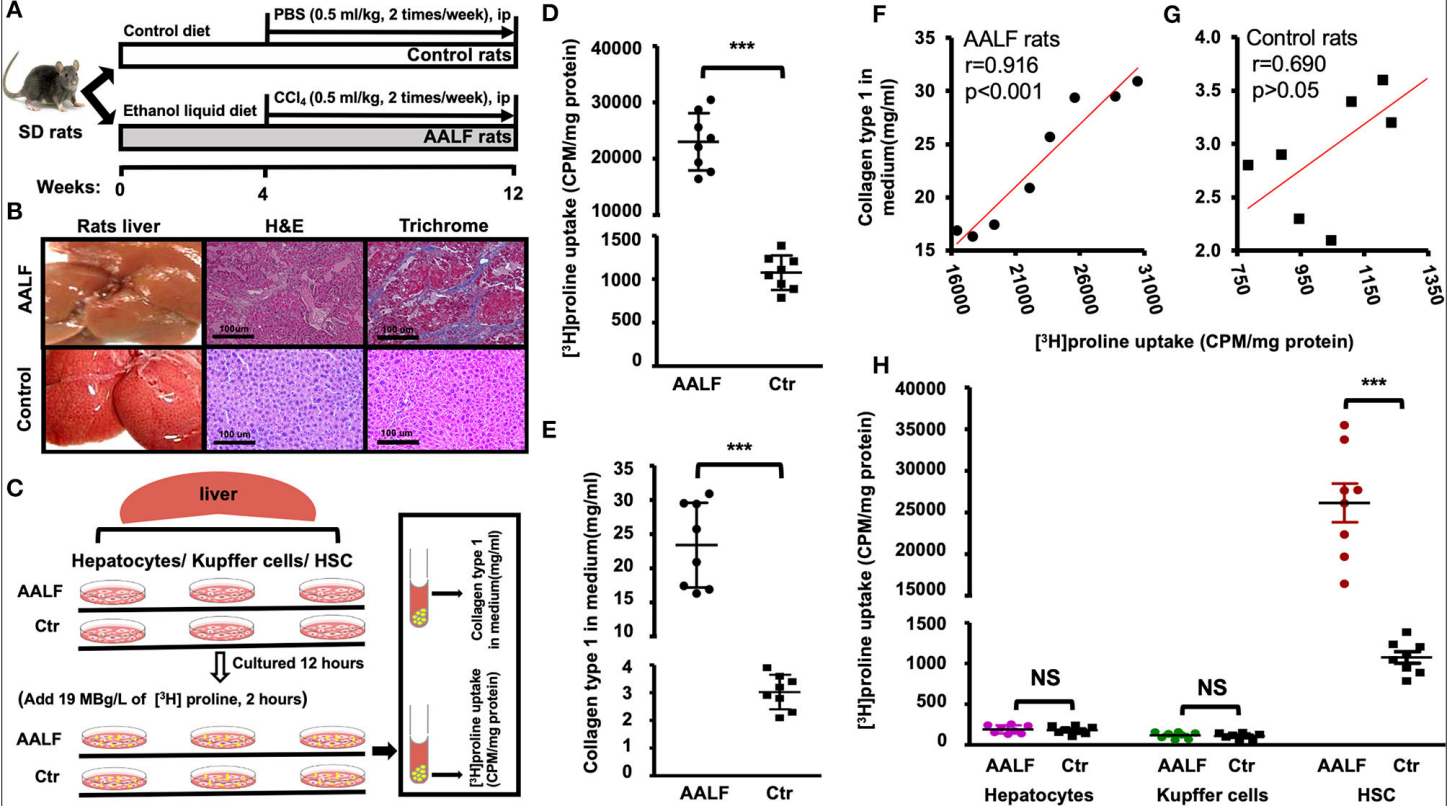
[^3^H]Proline uptake and collagen type 1 level in AALF rats and Controls. **(A)** The AALF rat model was induced by a modified Lieber-DeCarli liquid ethanol diet for 12 weeks along with intraperitoneal injection (IP) of CCl4 (0.5 ml/kg) during the 5th to 12th weeks, controls were fed an isocaloric liquid diet and IP. PBS. **(B)** Representative liver morphology, photomicrographs of H&E stain and trichrome stain of AALF and Control rats' liver. **(C)** [3H]Proline uptake by HSC isolated from livers was tested by a liquid scintillation counter, and collagen type 1 production in HSC culture medium was tested by Elisa. HSC, Kupffer cells, and hepatocytes isolated from AALF rats and controls were cultured in a medium for 12 h and then along with 19 MBg /L of [^3^H]Proline cultured for up to 120 min. **(D)** [^3^H]Proline uptake by HSC isolated from AALF rats' livers and controls (***p < 0.001, *n* = 8). **(E)** Pre-collagen type 1 concentration in HSC culture media from AALF rats and controls using ELISA (***p < 0.001, *n* = 8). **(F)** Correlation between [^3^H]Proline uptake and pre-collagen type 1 in AALF rats and data were derived from **(D,E)**. **(G)** Correlation between [^3^H]Proline uptake and pre-collagen type 1 in control rats and data were derived from **(D,E)**. **(H)**. [^3^H]Proline uptake by HSC, Kupffer cells, and hepatocytes from AALF rats and Controls. ***p < 0.001 or NS vs. controls. *n* = 8. CPM, counts per minute, AALF, advanced alcoholic liver fibrosis, Ctr, control, HSC, hepatic stellate cells, NS, no significant difference.

Schematic of the rat model (Figure 1A) has been described in the Methods section. Sections of rat livers are stained with hematoxylin and eosin (HE) to grade AALF activity (Figure 1B). Fibrotic collagen formation was seen in the AALF rats' livers and confirmed in sections stained with Masson's trichrome blue. In contrast, no significant fibrotic collagen deposits were seen in control liver sections. Trichrome stain showed fibrosis with numerous bridges or septae in the liver of AALF rats. In the liver tissue of controls, there is average collagen deposition around the portal tracts, while there is minimal deposition around the central veins. Histopathological analysis of livers from the AALF rats confirmed the presence of fibrosis which was not present in controls.

*In vitro* HSC study (Figure 1C) showed significant higher [^3^H]Proline uptake (23007.90 ± 5089.20 vs. 1075.40 ± 119.30 CPM/mg, *p* < 0.001, Figure 1D) in HSCs isolated from AALF rats than controls. Highly positive correlation between [^3^H]Proline uptake and collagen type 1 by HSC of AALF rats was found (*r* value = 0.91, *p* < 0.001, Figure 1F). There is no apparent relationship between the two variables by HSC of control rats (*r* value = 0.69, *p* > 0.05, Figure 1G). Collagen type 1 production in HSC culture medium had significant difference between two groups (24.30 ± 5.80 vs. 3.0 ± 0.62 mg/ml, *p* < 0.001, Figure 1E).

HSC, Kupffer cells, and hepatocytes were isolated from the livers of AALF rats and controls. [^3^H]proline was preferentially taken up by HSC compared to hepatocytes or Kupffer cells (Figure 1H). HSC isolated from the AALF rat livers (Figure 1H) demonstrated significantly higher [^3^H]proline uptake compared to controls (*p* < 0.001). In contrast, there was no significant difference in [^3^H]proline uptake by either hepatocytes or Kupffer cells isolated from the livers of AALF rats and their controls.

### Collagen type 1 and proline transporter protein expression in the livers of the AALF model and controls

**Figure 2:**
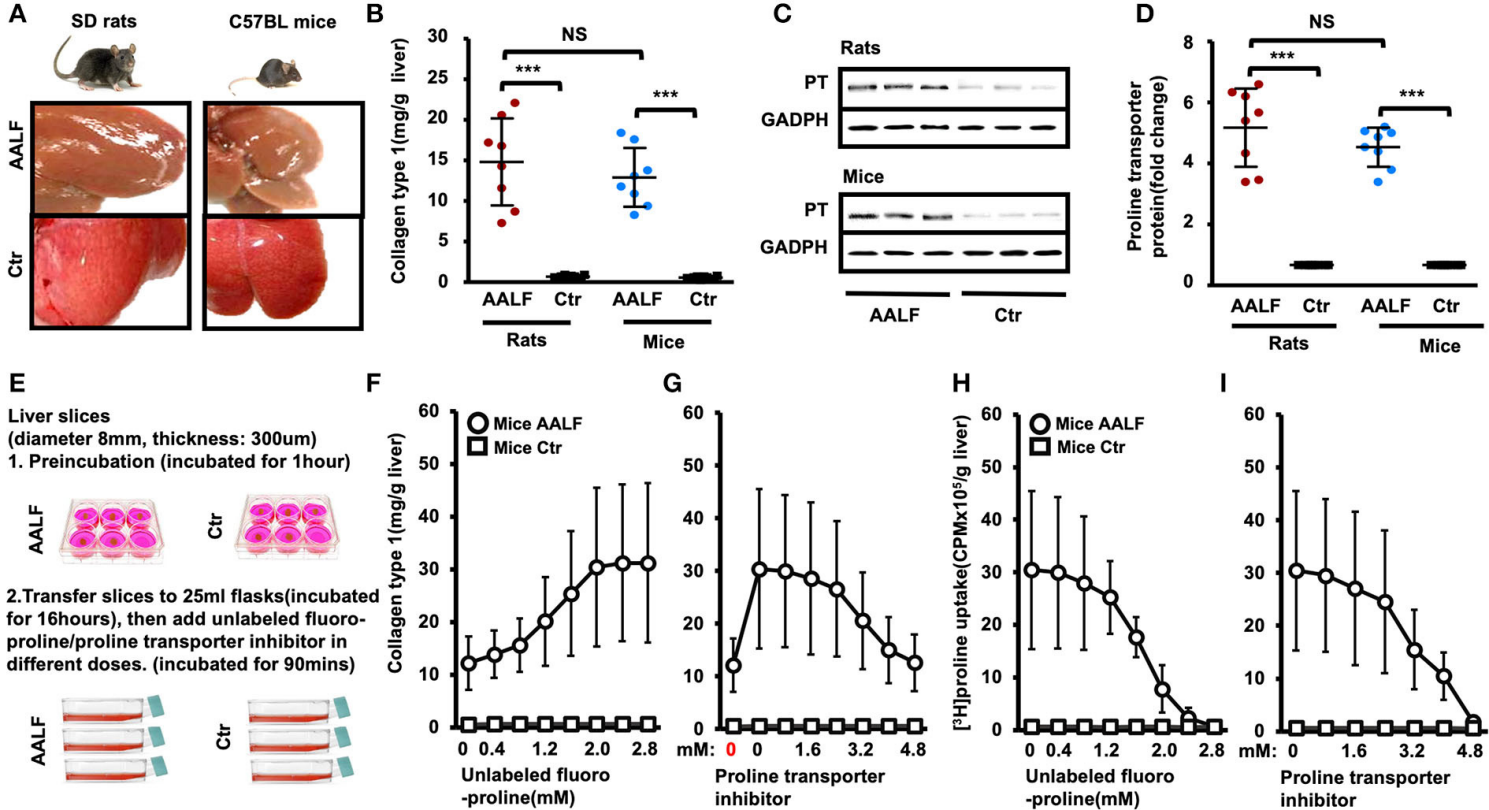
Collagen type 1 and proline transporter protein expression in the livers of experimental animal model and controls. [^3^H]Proline uptake *ex vivo* was tested using unlabeled proline and transporter inhibitor benztropine at different doses. **(A)** Representative liver morphology of AALF rats and AALF mice compared to corresponding controls. **(B)** Collagen type 1 in livers of AALF rats was compared with AALF mice and corresponding controls. ***p < 0.001 vs. corresponding controls. No significant difference between AALF rats and AALF mice. *n* = 8. **(C,D)** Proline transporter protein levels in livers of AALF rats and AALF mice and controls. ***p < 0.001 vs. corresponding controls. No significant difference between rats and mice with AALF. *n* = 8. **(E)** Experimental method for *ex vivo* liver slices. **(F)** Increased collagen synthesis in *ex vivo* liver tissues corresponding to different doses of unlabeled fluoro-proline in AALF mice compared with controls. **(G)** Inhibition of unlabeled fluoro-proline-stimulated liver collagen synthesis by the proline transporter inhibitor benztropine (0–4.8 mM) in AALF mice compared with controls in *ex vivo* liver tissue. *n* = 8 **(H)** *Ex vivo* liver [^3^H]proline uptake was inhibited competitively by unlabeled fluoro-proline in different doses in AALF mice compared with controls. *n* = 8. **(I)** *Ex vivo* liver [^3^H]proline uptake was inhibited specifically by the proline transporter inhibitor benztropine in AALF mice compared with controls. *n* = 8. PT, proline transporter, NS, no significant difference, AALF, advanced-stage alcoholic liver fibrosis, Ctr, control.

Schematic of the *Ex vivo* experiments (Figures 2A,E) has been described in the Methods section. [^3^H]proline uptake *ex vivo* was tested using unlabeled proline and transporter inhibitor benztropine at different doses. An *ex vivo* liver tissue study showed no significant difference in collagen type 1 levels between AALF rats and AALF mice (14.83 ± 5.35 mg/g vs. 12.91 ± 3.62 mg/g, *p* > 0.05) (Figure 2B). Proline transporter expression between AALF rats and AALF mice (7.76 ± 1.92-fold vs. 6.80 ± 0.97-fold) also had no significant difference between the two groups (Figures 2C,D). In this study, we found increased collagen synthesis in *ex vivo* liver tissues corresponding to different doses of unlabeled fluoro-proline in AALF mice compared with controls (Figures 2F,H). In *ex vivo* liver tissue, inhibition of unlabeled fluoro-proline-stimulated liver collagen synthesis by the proline transporter inhibitor benztropine (0-4.8 mM) in AALF mice compared with controls were shown (Figure 2G). *Ex vivo* liver [^3^H]Proline uptake was inhibited competitively by unlabeled fluoro-proline in different doses in AALF mice compared with controls (Figure 2I). An *ex vivo* liver [^3^H]proline uptake was inhibited explicitly by the proline transporter inhibitor benztropine in AALF mice compared with controls. Unlabeled fluoro-proline induced generation of liver tissue collagen type 1 and [^3^H]Proline uptake was specifically blocked by transporter inhibitor.

### Histopathology and blood biochemistry changes in AALF mice and controls

**Figure 3:**
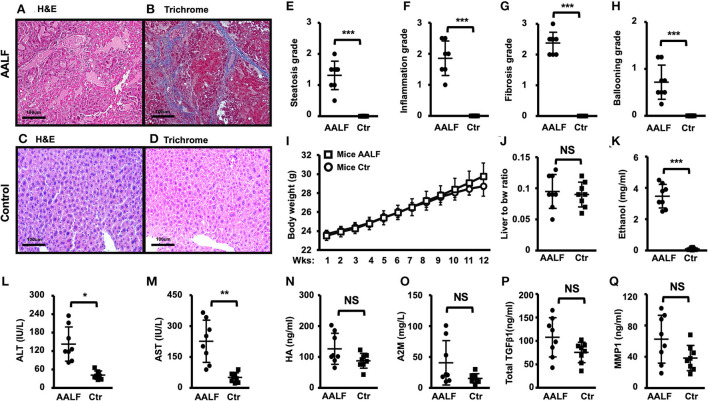
Histopathology and blood biochemistry changes in AALF mice and controls. **(A,B)** H&E and trichrome stain of livers of AALF mice. Fibrotic collagen deposition was seen in the livers of AALF mice stained with H&E, and fibrosis with numerous bridges or septae was confirmed in sections stained with Masson's trichrome blue. **(C,D)** H&E and trichrome stain of livers of normal controls. **(E–H)** Summary grades of steatosis **(E)**, inflammation **(F)**, fibrosis **(G)**, and ballooning **(H)** in livers of AALF mice, there were significant differences between the two groups. *n* = 8. **(I)** Body weight changes during the ethanol feeding periods of AALF mice vs. controls. *n* = 8. **(J)** The ratio of liver weights to body weights of AALF mice vs. controls. **(K)** Ethanol levels in AALF mice vs. controls. *n* = 8. **(L,M)** Two hepatic plasma enzymes, alanine aminotransferase (ALT), and aspartate aminotransferase (AST) in the serum of AALF mice vs. controls. *n* = 8. **(N,O)** Hyaluronic acid (HA) and alpha-2 macroglobulin (A2M) concentrations in mice with AALF mice vs. controls. *n* = 8. **(P,Q)** Total transforming growth factor-beta 1 (TGFβ1) and matrix metalloproteinase 1 (MMP1) concentrations in AALF mice vs. controls. *n* = 8. *p < 0.05, **p < 0.01, and ***p < 0.001 vs. corresponding controls. NS, no significant difference between AALF mice and controls. NS, no significant difference; AALF, advanced stage alcoholic liver fibrosis; Ctr, control.

Fibrotic collagen deposition was seen in the AALF mouse livers stained with H&E (Figure 3A), and fibrosis with numerous bridges or septae (Figure 3B) was confirmed in sections stained with Masson's trichrome blue. In contrast, no significant fibrotic collagen formation was seen in control liver sections (Figures 3C,D). Histopathological analysis of livers from the AALF mice confirmed the presence of fibrosis that was not present in control livers. It was similarly seen in the rats with AALF. Liver histology showed increased collagen deposition, summary grades of steatosis (Figure 3E), inflammation (Figure 3F), fibrosis (Figure 3G), ballooning (Figure 3H), and plasma ethanol levels in AALF mice were significantly higher than that of controls (Figure 3K). However, the variables of body weight (Figure 3I), the ratio of liver weights to body weights (Figure 3J), alanine aminotransferase (ALT) and aspartate aminotransferase (AST) in the serum, blood hyaluronic acid (HA) (Figure 3N) and alpha-2 macroglobulin (A2M) concentrations (Figure 3O), and total transforming growth factor-beta 1 (TGFβ1) (Figure 3P) and matrix metalloproteinase 1 (MMP1) concentrations (Figure 3Q) had no significant difference between AALF mice and controls.

### Static [^18^F]proline fused PET/CT imaging in AALF mice compared with controls

**Figure 4:**
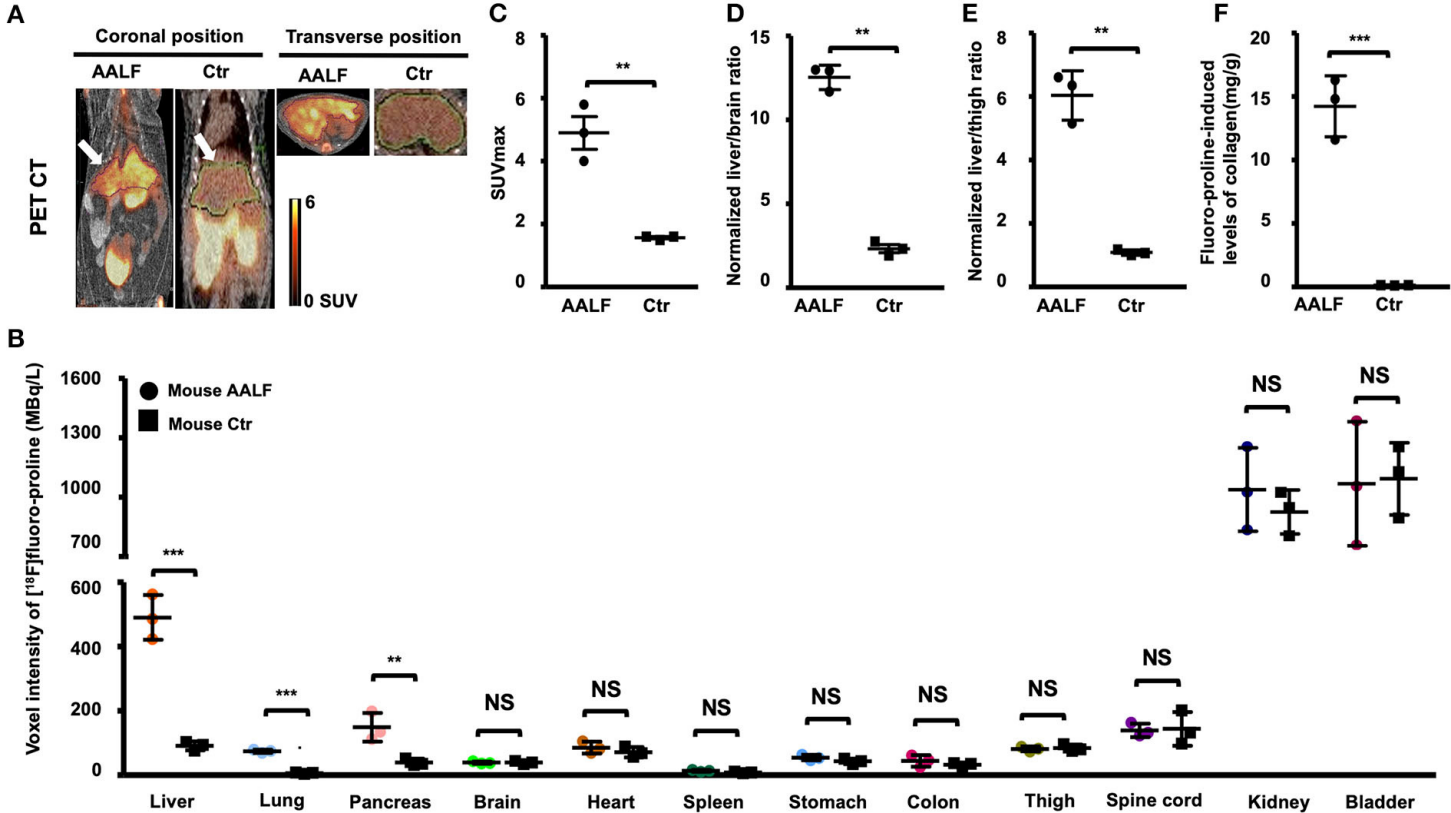
Static [^18^F]fluoro-proline fused PET/CT imaging in AAFL mice compared with controls. **(A)** Coronal and transverse views of [^18^F]proline positron emission tomography/computed tomography (PET/CT) imaging in AALF mice vs. controls. The white arrow indicates the livers of the animals. Images were performed 60 min after [^18^F]Proline was administrated via the tail vein, and total imaging length was acquired for 30 min. The representative mouse body weights were 30 g for the control mouse and 29 g for the AALF mouse, and the injected doses were 12 MBq, respectively, of [^18^F]proline at the time of imaging experiments. **(B)** Voxel intensity of [^18^F]proline activity in liver, lung, pancreas, and other 9 organs of AALF mice vs. controls. *n* = 3. **(C)** [^18^F]proline activity in SUV max in the livers of AAFL mice vs. controls. **p < 0.01 vs. controls. **(D)** Normalized liver to brain ratio in SUVmax of AAFL mice vs. controls. **p < 0.01 vs. controls. **(E)** Normalized liver to thigh muscle ratio in SUVmax of AAFL mice vs. controls. **p < 0.01 vs. controls. **(F)** Fluoro-proline-induced levels of collagen in *ex vivo* liver tissues in AAFL mice vs. controls. ***p < 0.001 vs. controls. NS, no significant difference; AALF, advanced stage alcoholic liver fibrosis; Ctr, control.

*In vivo* [^18^F]proline micro PET/CT imaging showed higher SUVmax in liver (4.90 ± 0.91 vs. 1.63 ± 0.38, *p* < 0.01) (Figures 4A,C), higher normalized liver/brain ratio (12.54 ± 0.72 vs. 2.33 ± 0.41, p < 0.01) (Figure 4D), and higher normalized liver/thigh ratio (6.03 ± 0.78 vs. 1.09 ± 0.09, *p* < 0.01), which are all positive correlated with fluoro-proline-induced levels of collagen in liver tissue (r value ≥ 0.93, *p* < 0.01), but not with existing liver collagen in AALF mice and controls.

There was a significant increase in [^18^F]proline activity in the liver of AALF mice compared with control mice (*p* < 0.001, Figure 4B). There was also increased uptake in the lungs and pancreas of AALF mice (*p* < 0.001 and *p* < 0.01) compared with control mice (Figure 4B). There was no statistically significant difference in [^18^F]proline activity in the other nine organs (brain, heart, spleen, kidneys, bladder, stomach, colon, thigh, and spinal tube fibrotic components). Correlation among liver collagen, liver SUVmax, normalized liver to brain ratio, normalized liver to thigh ratio, and total liver collagen after fluoro-proline-inducing or fluoro-proline-induced levels of collagen in *ex vivo* liver tissues in AALF mice.

**Figure 5:**
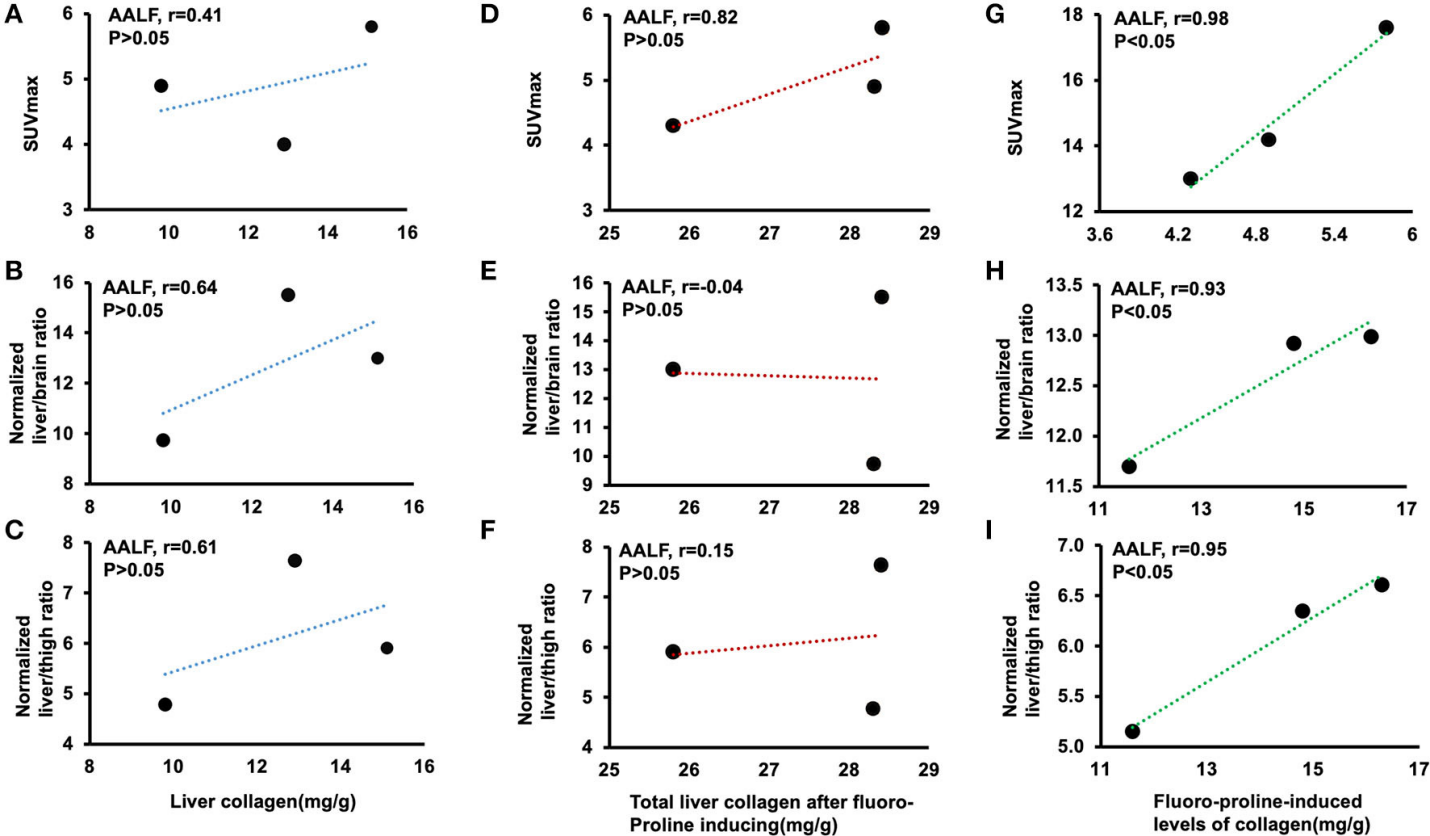
Correlation among liver collagen, liver SUVmax, normalized liver-to-brain ratio, normalized liver-to-thigh ratio, and fluoro-proline-induced collagen levels in *ex vivo* liver tissues in AALF mice. **(A–C)** Correlation between liver collagen and liver SUVmax, normalized liver to brain ratio, and normalized liver to thigh ratio in AALF mice, respectively. *n* = 3. **(D–F)** Correlation between total liver collagen in *ex vivo* liver tissues after fluoro-proline-induced and liver SUVmax, normalized liver to brain ratio, and normalized liver to thigh ratio in AALF mice, respectively. *n* = 3. **(G–I)** Correlation between fluoro-proline-induced levels of collagen in *ex vivo* liver tissues and liver SUVmax, normalized liver to brain ratio, and normalized liver to thigh ratio in AALF mice. *n* = 3. Fluoro-proline-induced collagen levels in *ex vivo* liver tissues = total liver collagen in *ex vivo* liver tissues after fluoro-proline-induced- liver collagen in *ex vivo* liver tissues. AALF, advanced-stage of alcoholic liver fibrosis.

There is no statistical evidence for a linear relationship among correlation between liver collagen and liver SUVmax (*r* = 0.41, *p* > 0.05), normalized liver to brain ratio (*r* = 0.64, *p* > 0.05), and normalized liver to thigh ratio (*r* = 0.61, *p* > 0.05) in *ex vivo* liver tissues in AALF mice (Figures 5A–C). Total liver collagen in *ex vivo* liver tissues after fluoro-proline-inducing also had no apparent correlation among the three groups (Figures 5D–F). Fluoro-proline-induced levels of collagen in *ex vivo* liver tissues and liver SUVmax (*r* = 0.98, *p* < 0.05), normalized liver to brain ratio (*r* = 0.93, *p* < 0.05), and normalized liver to thigh ratio (*r* = 0.95, *p* < 0.05) have a statistical evidence for a linear relationship among the pairs of variables in AALF mice (Figures 5G–I).

## Discussion

Here, we observed a remarkable correlation between liver maximum standard uptake value (SUVmax) on PET/CT imaging and Fluoro-proline-induced levels of liver collagen. The uptake of [^18^F]proline was not associated with the collagen deposited in the liver and the total hepatic collagen content. This result is probably explained by the fact that the marker is specifically targeting active collagen. Our study suggests that PET/CT with [^18^F]proline has the potential sensitivity to reflect the liver collagenogenesis and assess the activity and severity of liver fibrosis.

CCl4 induction was the most commonly used strategy to generate liver fibrosis in animal model (19). After intratracheal instillation of CCl4 for 8 weeks, high-density lipoprotein (HDL) and total protein decreased remarkably, while liver enzymes and oxidative stress markers increased dramatically (11). The proliferation of fibroblasts and excessive accumulation of extracellular matrix occurred at the later phase of this disease model. Prominent collagen deposition and fiber segmentation formation was detected histologically and biochemically. Masson trichrome staining helped to confirm the pathologic process of liver fibrosis. Liver tissues of the CCL4 group showed histologic modifications, including over-proliferation of fibroblasts and excessive deposition of collagen and extracellular matrix proteins. According to histologic staining, steatosis, inflammation, fibrosis, and ballooning grade of the CCL4 group were significantly higher than that of the control group. These data confirmed that the CCL4-induced rat liver fibrosis model was successfully established, and a solid fibrotic response occurred at the later period of this disease model.

Collagen protein synthesis was increased in hepatic stellate cells activation of AALF animal models and associated with the degree of fibrosis (20). Hepatic stellate cells are liver-specific mesenchymal cells that play vital roles in the progression of ALD to liver fibrogenesis (17, 21, 22). As fibrosis advances, the collagenous bands typical of cirrhosis contain large numbers of activated stellate cells (23). Proliferative activity and collagen-stimulating activity was determined by [^3^H]Proline incorporation assay using rat HSC. Collagen type 1 is a heterotrimer consisting of three chains with a helical region made of repeating units of proline and hydroxyproline (24). [^3^H]Proline uptake was seen predominantly in HSCs isolated from acute-on-chronic steatohepatitis livers, as opposed to liver Kupffer cells and hepatocytes (15). *In vitro* and *ex vivo* culture systems, [^3^H]proline uptake was used to evaluate the effects of the activation of hepatic stellate cells and their role in the development of collagen protein synthesis.

Recent studies reported that 18F-fluorodeoxyglucose ([^18^F]FDG) PET/CT could be used for molecular imaging of liver fibrosis in animal models and humans (13, 25). [^18^F]FDG is the most common radiotracer in use today. Hernandez-Martinez Ana et al. reported that the [^18^F]FDG uptake in cirrhotic livers was reduced compared to that in the non-cirrhotic livers (26). However, Shu Su et al. proposed that [^18^F]FDG PET/CT could not be applied to monitor the progression of liver fibrosis, whereas Al[^18^F]F-NOTA- PEG3-Duramycin PET/CT could (27). Therefore, the necessity of obtaining a specific PET/CT imaging marker for liver fibrosis remains controversial. We had previously found that [^18^F]proline correlates with collagenogenesis in acute steatohepatitis in the liver using micro-PET/CT imaging (15). In this study, we used the AALF mice model and found that the high uptake of [^18^F]proline was more associated with fluoro-proline-induced levels of collagen in liver tissue instead of the collagen already deposited in the liver. These findings align with prior biodistribution studies: PET/CT imaging of pulmonary fibrosis with 2-(5-[^18^F]fluoropentyl)-2-methyl malonic acid in a rabbit model indicated sensitive and specific identification of silicosis in early stages (12). High uptake of radiotracer seemed to be more associated with an early inflammatory phase of pulmonary fibrosis as a peak of uptake, decreased during the later fibrotic stage (12, 28). Based on these insights, PET/CT may be a potential method for evaluating fibrotic activity and staging. Our study suggests that PET/CT with [^18^F]proline can be used to target active collagen specifically instead of existing liver collagen.

PET/CT studies of whole human body distribution showed retention of [^18^F]proline in the liver, lung, pancreas, and renal cortex in agreement with its acceptance for protein biosynthesis (29, 30). We found significantly higher [^18^F]proline uptake in the liver, lung, and pancreas compared with its normal controls, and remarkably higher intracellular a1 procollagen mRNA expression in the HSCs of acute-on-chronic steatohepatitis mice with liver fibrosis compared with those of control mice (15). A more significant increase in collagen synthesis from baseline levels was found when the liver tissues of AALF mice were incubated with proline compared with those of the control mice, but less [^3^H]proline was taken up as the concentration of unlabeled fluoro-proline increased, suggesting that the mechanism of proline uptake in the liver is saturable (12). It is interesting to note that proline transporter inhibitors could bring proline-increased collagen synthesis back to baseline levels, with an associated decreased [^3^H]proline uptake (31). Our data proposed that [^18^F]proline uptake of liver tissues increased significantly at the early inflammatory phase and slowly decreased at the late fibrotic phase of mouse liver fibrosis, which was well aligned with findings in previous animal [^18^F]FDG PET/CT studies (32). These data mentioned above helped confirm the induction of strong fibrotic activity during the progression of this disease in a rat model. Therefore, molecular imaging of intake of [^18^F]proline may have essential implications for the non-invasive diagnosis of liver fibrosis and evaluation of the fibrotic activity. Furthermore, no correlation was observed between [^18^F]FDG uptake of liver tissues and fibrotic activity. Compared with [^18^F]FDG PET/CT, our study suggested that PET/CT with [^18^F]proline could better reflect the fibrotic process of this disease and assess the severity and activity of liver fibrosis.

Our experiment had certain limitations. This study was performed in a CCL4-induced liver fibrosis mouse model, which might not reflect all types of liver fibrosis. Therefore, further studies of PET/CT imaging with [^18^F]proline to other kinds of liver fibrosis models and clinical trials would be required to demonstrate further the capabilities of [18F]proline for liver fibrosis imaging. Comparison of PET SUVmax value by liver region was less exact in this small animal model. This may reflect the limited resolution of the clinical PET scanner used in our study in this relatively small animal model. While the mouse's liver is of sufficient size to resolve PET features between the general liver and non-liver field, the liver lobes cannot be distinguished precisely, and features near the mediastinum sometimes cannot be definitively localized in the images. It should also be mentioned that the ongoing cycles of injury and repair that lead to the accumulation of scar tissue are similar whether it occurs in response to chronic injury of the liver or other organs like the kidney, lungs, Cardiac, renal, pancreas, and pulmonary fibrosis is characterized by excess deposition of type I collagen. Therefore, we calculated the normalized liver-to-brain ratio and the normalized liver-to-thigh muscle ratio in SUVmax value of AALF mice to conduct a more appreciative evaluation.

## Conclusion

The [^18^F]proline micro PET/CT cannot be used to quantify existing liver collagen in AALF mice but may be used for evaluating collagenogenesis in activated HSC of experimental AALF. [^18^F]proline PET/CT may be a potential method for diagnosing early-stage liver fibrosis, evaluating fibrotic activity, and assessment of the efficacy of antifibrotic treatments in clinical studies.

## Data availability statement

The original contributions presented in the study are included in the article/supplementary materials, further inquiries can be directed to the corresponding author/s.

## Ethics statement

The animal study was reviewed and approved by Institutional Animal Care and Use Committee of University of Maryland School of Medicine. Radiation Safety Operation Committee of University of Maryland School of Medicine.

## Author contributions

ND wrote the paper. QC designed the research. HC performed the research and contributed equally to this manuscript. LP and YA analyzed the data. ND, HC, LP, YA, and QC reviewed the manuscript. All authors contributed to the article and approved the submitted version.

## Funding

This work was in part supported by research grants from the National Institute on Alcohol Abuse and Alcoholism of the National Institutes of Health (Award Number K08AA024895-QC and R01AA028995-01-LP/sub-PI-QC), and the Chair Research Foundation of the University of Maryland School of Medicine Department of Diagnostic Radiology and Nuclear Medicine (QC).

## Conflict of interest

The authors declare that the research was conducted in the absence of any commercial or financial relationships that could be construed as a potential conflict of interest.

## Publisher's note

All claims expressed in this article are solely those of the authors and do not necessarily represent those of their affiliated organizations, or those of the publisher, the editors and the reviewers. Any product that may be evaluated in this article, or claim that may be made by its manufacturer, is not guaranteed or endorsed by the publisher.

## Abbreviations

AALF: advanced-stage alcoholic liver fibrosis
HSC: hepatic stellate cells
SD: Sprague-Dawley
CCL4: C-C Motif Chemokine Ligand 4
ELISA: enzyme linked immunosorbent assay
qRT-PCR: quantitative real-time polymerase chain reaction
H&E: hematoxylin and eosin
MBq/L: megabecquerel per liter
ALT: alanine aminotransferase
A2M: alpha-2-macroglobulin
AST: aspartate amino transferase
HA: hyaluronic acid
TGFβ1: total transforming growth factor beta 1
MMP1: matrix metalloproteinase 1
CPM: counts per minute
[^18^F]proline: cis-4-[^18^F]fluoro-L-proline
[^18^F]FDG,: ^18^F-fluorodeoxyglucose PET/CT, positron emission tomography/computed tomography
SUV: standardized uptake value.
